# Nogapendekin alfa Inbakicept-pmln (Anktiva) with BCG: A Promising Arsenal in BCG-Unresponsive Non-Muscle-Invasive Bladder Cancer Intervention

**DOI:** 10.34172/apb.43870

**Published:** 2025-03-29

**Authors:** ArunSundar MohanaSundaram, Velmurugan Raja, Yeshwanth Kamalakannan, Md Aminul Islam

**Affiliations:** ^1^School of Pharmacy, Sathyabama Institute of Science and Technology, Jeppiaar Nagar, Rajiv Gandhi Salai, Chennai 600119. Tamilnadu, India.; ^2^COVID-19 Diagnostic Lab, Department of Microbiology, Noakhali Science and Technology University, Noakhali-3814, Bangladesh.

## Dear Editor,

 Bladder cancer (BC), the 9^th^ most diagnosed cancer worldwide, is associated with high recurrence rates and substantial lifetime treatment costs. Non-muscle-invasive bladder cancer (NMIBC) accounts for 75% of new cases and transurethral resection of bladder tumor (TURBT) remains the preferred treatment modality, followed by intravesical bacillus Calmette-Guérin (BCG).^1^ However, 20% of high-risk NMIBC cases are BCG-unresponsive, necessitating radical cystectomy, a procedure that is often associated with significant morbidities and poor quality of life. This finding highlights the critical need for novel bladder-sparing therapies. Currently available U.S. Food and Drug Administration (FDA)-approved chemotherapeutics/immunotherapeutics (e.g., valrubicin, pembrolizumab, and nadofaragene firadenovec-vcng) only mitigate the progression to muscle-invasive BC rather than accomplish disease control in BCG-unresponsive HR-NMIBC cases.^1^

 Interestingly, the recent FDA-approved therapy ANKTIVA (N-803; ALT-803; nogapendekin alfa inbakicept-pmln; NAI) along with BCG seems to offer promising outcomes in BCG-u NMIBC patients.^2^ The immunopharmacological mechanism of N-803 admixed with BCG is a complex process involving the stimulation of the innate and adaptive immune systems. Intravesical BCG selectively binds to urothelial and tumor cells in the bladder via fibronectin. BCG attenuates antitumor immune responses through HLA-1 downregulation and activates the tumor clearance process by triggering a complex pro-inflammatory response and stimulating natural killer (NK) cells and CD8 + T cells. N-803, an IL-15 superagonist/IL-15RαSushi-Fc fusion complex, promotes the activation and proliferation of local immune cells, including CD4 + and CD8 + T cells, NK cells, and memory T cells, and has potent antitumor activity.^3,4^

 In a Phase2/3 clinical trial, approximately 71% of patients treated with N-803 admixed with BCG achieved complete response (CR) in 26.6 months (median duration), while CR at 12-months was 45%.^3^ In the same cohort, 86% of the patients reported grade 1 or 2 adverse events (AEs) (Table 1). Clinical evidence of alternative therapeutics indicates that pembrolizumab, nadofaragene-firadenovec, gemcitabine/docetaxel, and valrubicin showed 46%, 24.3%, 54%, and 18% CR respectively at 12-month.^5,6^ Approximately 66% of the subjects treated with pembrolizumab reported AEs, including fatigue, diarrhea, and pruritus. Approximately 70% of patients treated with nadofaragene-firadenovec reported AEs, with nearly 4% presenting with grade 3 or more AEs. Nearly 70% of the patients treated with valrubicin showed local bladder symptoms during treatment.

**Table 1:** An overview of the clinical trial details of N-803

**Clinical trial**	**Sample size**	**Methodology**	**Duration and outcome**	**Treatment-related adverse events**
Phase 1b^7^ (NCT02138734)	9	This open-label dose-escalation trial included intermediate- or high risk, BCG-naïve, NMIBC patients with a recent history* of TURBT, diagnostic biopsy, and cystoscopy. The participants were divided into three cohorts. Intravesical BCG, 50 mg/instillation in combination with increasing doses of N-803 in three cohorts (100, 200, or 400 µg N-803 per instillation). The median age of the participants in each cohort is 70, 75, and 65 years respectively. This study included patients with histologically confirmed NMIBC (Ta or T1, or carcinoma in situ (CIS)), who were at intermediate or high risk of disease and BCG-naïve (i.e., who had not received prior treatment with BCG). Safety and tolerability of BCG + N-803 were the primary end-points.	After six years of post-treatment follow-up, all the participants remained disease-free. This combination was well-tolerated; The recommended dose for phase 2 was fixed at 400 µg N-803 per instillation.	No dose-limiting toxicity was observed. Hypertension was the most common treatment-related adverse event (TRAE) observed in 67% of patients. Other TRAEs included fatigue, hematuria, and urinary frequency.
Phase 2/3^3^ (NCT03022825)	164	This open-label, multicenter trial included histologically confirmed NMIBC patients, aged 18 years or older, under three cohorts: Cohort A (median age: 73 years): BCG-unresponsive CIS with or without Ta/T1 carcinoma (treated with BCG + N-803); Cohort B (median age: 72 years): BCG-unresponsive high-grade papillary disease (treated with BCG + N-803); Cohort C (median age: 74.5 years): BCG-unresponsive CIS with or without Ta/T1 carcinoma (treated with N-803 only). This study included NMIBC patients with histologically confirmed high-grade carcinoma in situ (CIS) with or without Ta/T1 papillary disease, and who were unresponsive to BCG, indicating a more resistant stage of disease progression. The primary endpoint was durable complete responses (CR).	In Cohort A, 71% of the patients achieved CR at 26.6 months, Disease-specific survival was 100% at 24 months. In Cohort B, Disease-specific survival was 97.7% at 24 months. In the Cohort C, CR was achieved in only 20% (2/10) of patients at 3 months and in one patient at 6 months. The remaining patients underwent re-induction.	In both cohorts A and B, the most common TEAE, primarily due to BCG instillation, included dysuria, pollakiuria, and hematuria. The incidence of grade 1 or 2 TEAEs was 86%, and those of grades 3, 4, and 5 TEAEs were 20%, 2%, and 1%, respectively. In Cohort C, only one patient (out of 10) experienced grade 3 stroke. Approximately 70% of patients presented with only grade 1 or 2 TEAEs.

*Recent history indicates TURBT and bladder biopsy within 3 months before the study and a pre-study cystoscopy within one month.

 Although N-803 exhibits a good efficacy and safety profile, the high cost of N-803 and BCG shortage hinder the affordability and accessibility of this therapy. Several countries encounter shortages of BCG due to manufacturing and logistic constraints and the consequent increase in cost amidst high demand. BCG shortages can be mitigated through various strategies, such as improving global production (through government incentives, enhancing local production, promoting collaboration with current manufacturers), improving logistics (e.g., digital tracking), developing cost-effective substitutes (e.g., alternative intravesical therapies and novel immunotherapies), and preventing stockpiling and reducing BCG waste (e.g., split-dose regimens).^8^ In low-resource settings, prioritizing N-803/BCG administration in high-risk cases and induction therapy over low-risk/maintenance therapy may be effective.

 The estimated cost of N-803 is approximately USD 100 000-150 000 for a complete treatment course, which makes the treatment expensive and less accessible.^9^ The total treatment costs (over a 10-year horizon) for gemcitabine/docetaxel and pembrolizumab are USD 50 000 and USD 210 000, respectively.^10^ A recent study by D’Andrea et al. found that, in terms of the incremental cost-effectiveness ratio (ICER) per quality-adjusted life year (QALY), N-803 (ICER: 44,602 USD) demonstrated lower cost-effectiveness than nadofaragene firadenovec (ICER: 10,014 USD).^11^ Although the ICER value for pembrolizumab was unspecified, they underscored that this treatment is less costly and more effective than the other two options. Some experts consider gemcitabine/docetaxel as an affordable standard of care for managing BCG-u NMIBC.^12^ However, its efficacy remains to be established through prospective clinical trials. Taken together, various prospective studies on the long-term clinical efficacy (e.g., disease-free survival at 5 years post-treatment), safety profile (e.g., potential late-onset adverse events), comparative trials with other intravesical chemotherapies, and cost-effective dosage regimens of N-803 in resource-constrained settings, amidst BCG shortages, are necessary to optimize treatment strategies, mitigate AEs, and ensure cost-effectiveness.

 In addition to the regulatory approval of ANKTIVA (N-803) with BCG for BCG-u-NMIBC patients, our communication provides a multifaceted viewpoint on comparative data on the efficacy, safety, and cost-effectiveness of N-803 and other treatment modalities. In addition, various insights into overcoming the BCG shortage and utilization of N-803 in low-resource settings have been discussed. Although this therapy holds potential as a future standard of care, accessibility and affordability are looming concerns. However, it offers a ray of hope for a subset of patients with NMIBC who would otherwise undergo highly invasive surgery with enduring repercussions.

## Conclusion

 The U.S. The FDA approval of ANKTIVA (N-803) in combination with BCG is a novel therapeutic arsenal for patients with BCG-unresponsive NMIBC. With robust clinical data on complete response rates and safety profiles, this immunotherapy regimen may serve as a bladder-sparing alternative to radical cystectomy. Nevertheless, the high cost of N-803 and the shortage of BCG hinder the widespread adoption of this therapy. To combat these issues, a multifaceted approach involving supply chain optimization, policy reforms (e.g., drug pricing and reimbursement), and additional research into cost-effective application strategies is necessary. As the clinical evidence base continues to expand, this therapeutic approach has the potential to redefine the standard of care for a patient population that has long faced limited and high-risk treatment modalities.

## Competing Interests

 None.

## Ethical Approval

 Not applicable.
